# MSC microvesicles loaded G-quadruplex-enhanced circular single-stranded DNA-9 inhibits tumor growth by targeting MDSCs

**DOI:** 10.1186/s12951-024-02504-6

**Published:** 2024-05-12

**Authors:** Jingxia Han, Rong Qin, Shaoting Zheng, Xiaohui Hou, Xiaorui Wang, Huihui An, Zhongwei Li, Yinan Li, Heng Zhang, Denghui Zhai, Huijuan Liu, Jing Meng, Tao Sun

**Affiliations:** 1 State Key Laboratory of Medicinal Chemical Biology and College of Pharmacy, Nankai University, Tianjin, China; 2 State Key Laboratory of Food Nutrition and Safety, Tianjin University of Science and Technology, Tianjin, China; 3 Precision Medicine Center, The First Affiliated Hospital of Xi’an Jiaotong University, Xi’an, China; 4 College of Life Sciences, Nankai University, Tianjin, China

**Keywords:** Myeloid-derived suppressor cells, G-quadruplex-enhanced circular single-stranded DNA-9, miR-9, Umbilical cord mesenchymal stem cells, Melanoma

## Abstract

**Background:**

Myeloid-derived suppressor cells (MDSCs) promote tumor growth, metastasis, and lead to immunotherapy resistance. Studies revealed that miRNAs are also expressed in MDSCs and promote the immunosuppressive function of MDSCs. Currently, few studies have been reported on inducible cellular microvesicle delivery of nucleic acid drugs targeting miRNA in MDSCs for the treatment of malignant tumors.

**Results and conclusion:**

In this study, we designed an artificial DNA named G-quadruplex-enhanced circular single-stranded DNA-9 (G4-CSSD9), that specifically adsorbs the miR-9 sequence. Its advanced DNA folding structure, rich in tandem repeat guanine (G-quadruplex), also provides good stability. Mesenchymal stem cells (MSCs) were prepared into nanostructured vesicles by membrane extrusion. The MSC microvesicles-encapsulated G4-CSSD9 (MVs@G4-CSSD9) was delivered into MDSCs, which affected the downstream transcription and translation process, and reduced the immunosuppressive function of MDSCs, so as to achieve the purpose of treating melanoma. In particular, it provides an idea for the malignant tumor treatment.

**Graphical Abstract:**

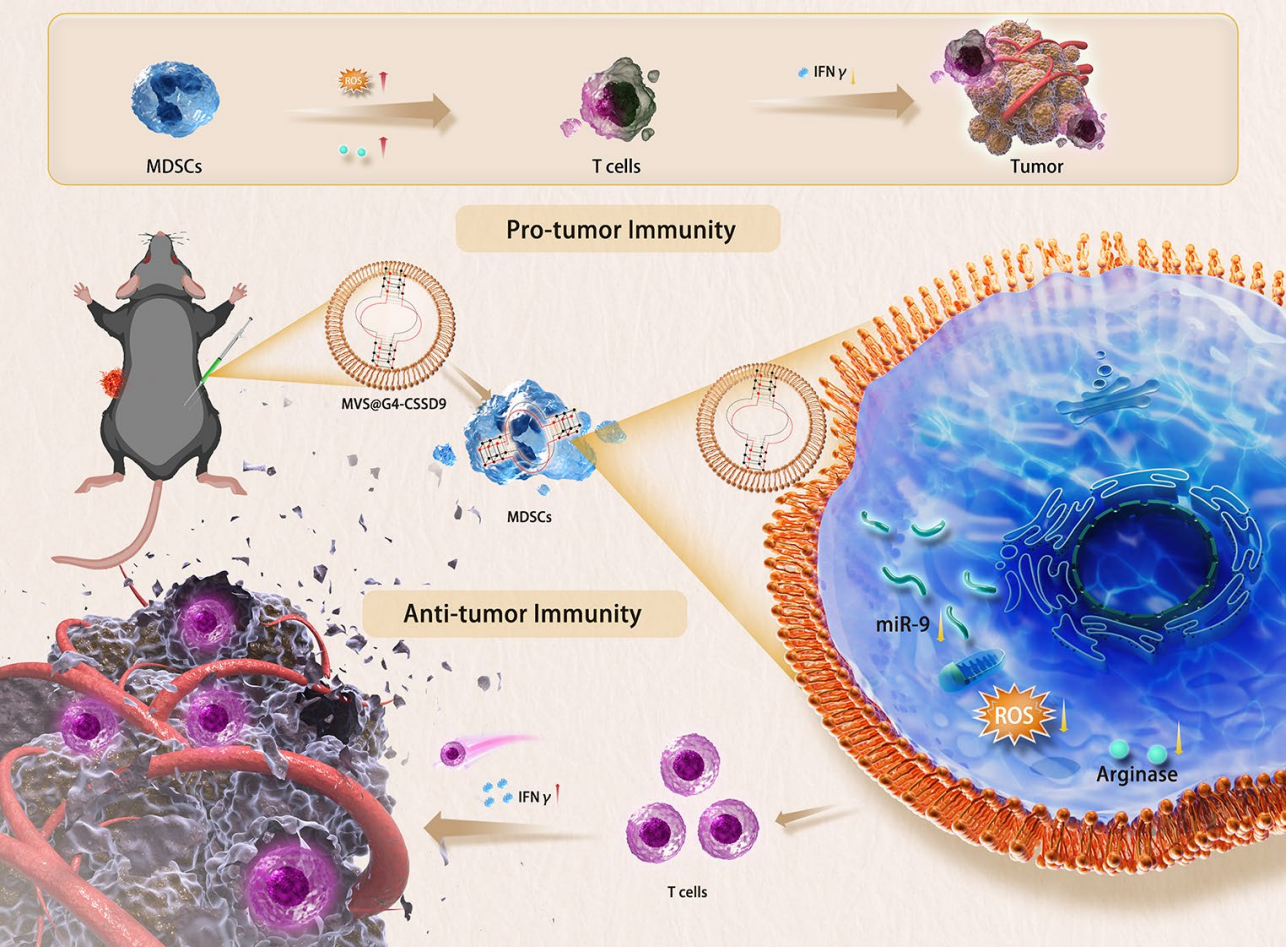

**Supplementary Information:**

The online version contains supplementary material available at 10.1186/s12951-024-02504-6.

## Introduction

MDSCs, defined as cells expressing CD11b and Gr-1, have been demonstrated to inhibit the proliferation and activation of T cells [1, 2]. MDSCs act to suppress T cell function through a number of mechanisms involving arginase 1, NO synthase, and reactive oxygen species [3]. MDSCs are major players in the orchestration of an immunosuppressive network in many pathologic conditions, such as chronic inflammation and cancer [1, 4–7]. Suppression of MDSCs is important for improving the immune microenvironment and the treatment of tumors. miRNAs play crucial roles in the regulation of diverse biological processes, including tumorigenesis and inflammation [8, 9]. Many studies have been shown that miRNAs regulate MDSCs maturation, differentiation and function [10]. Therefore, regulating the levels of miRNAs in MDSCs may modulate the immunosuppressive microenvironment of tumors and inhibit their malignant evolution. In order to explore the miRNAs that play a major role in MDSCs. In this study, miRNAs expressed in MDSCs and affecting their functions were screened using the GEO database and combined with experimental validation, based on which further anti-tumor nanomedicine design was carried out.

Circular RNA has the role of specific adsorption of miRNAs. In our previous study, circular single-stranded DNA-9 (CSSD9), which is rich in miRNA binding sites, was developed to adsorb miR-9 and in turn rescind the co-silencing of oncogenes present in some of the tumors and exerts an anti-tumor effect [11]. G-quadruplex are four-stranded DNA secondary structures that are deviated from the normal duplex form of DNA [12]. They comprise Hoogsteen hydrogen-bonded guanine quartet motifs formed from the guanine tracts in these sequences, together with intervening sequences which form extrahelical loops and help to hold together the G-quartets [13–15]. G-rich oligomers comprise a large group of aptamers with the ability to fold into stable G4 structures. It has better stability than circular RNA and is not easily degraded. To further improve the stability and activity of CSSD9, G-quadruplex was introduced in this study and G4-CSSD9 was prepared in the hope of further improving the stability and biological activity of CSSD9.

Extracellular vesicles (EVs) are cell-derived lipid bilayer-enclosed entities, secreted by many cell types. As nano-carries, it plays a significant role in delivery of proteins and nucleic acids [16, 17]. Compared with liposomes, micelles, and polymeric nanoparticles, EVs have many advantages as a natural carrier. It can evade phagocytosis and has natural targeting capacity. However, the yield of EVs collected from cultured cell supernatants is low in the current study. Therefore, the vesicles with a significantly greater yield are worth for the development of future nano-sized drug delivery. Extracellular vesicles are prepared by subjecting MSCs to serial extrusion through filters with diminishing pore sizes. MSCs have immunomodulatory potential and low immunogenicity [18, 19]. Both MSCs and MDSCs are closely related to immune function, within which there may be a natural targeting between them. To improve the delivery efficiency of G4-CSSD9, the present study used UC-MSCs EVs as a delivery vehicle to target MDSCs. and the present study also explored methods to increase the production of cellular microvesicles (MVs).

Based on the above research background, in this study, microvesicles (MVs) were obtained from induced MSCs by pressure extrusion, and then MVs@G4-CSSD9 were prepared and their effects on MDSCs and their antitumor effects were investigated.

## Results

### MiR-9 could contribute to MDSCs induction and immunosuppressive function in vitro.

**Fig. 1:**
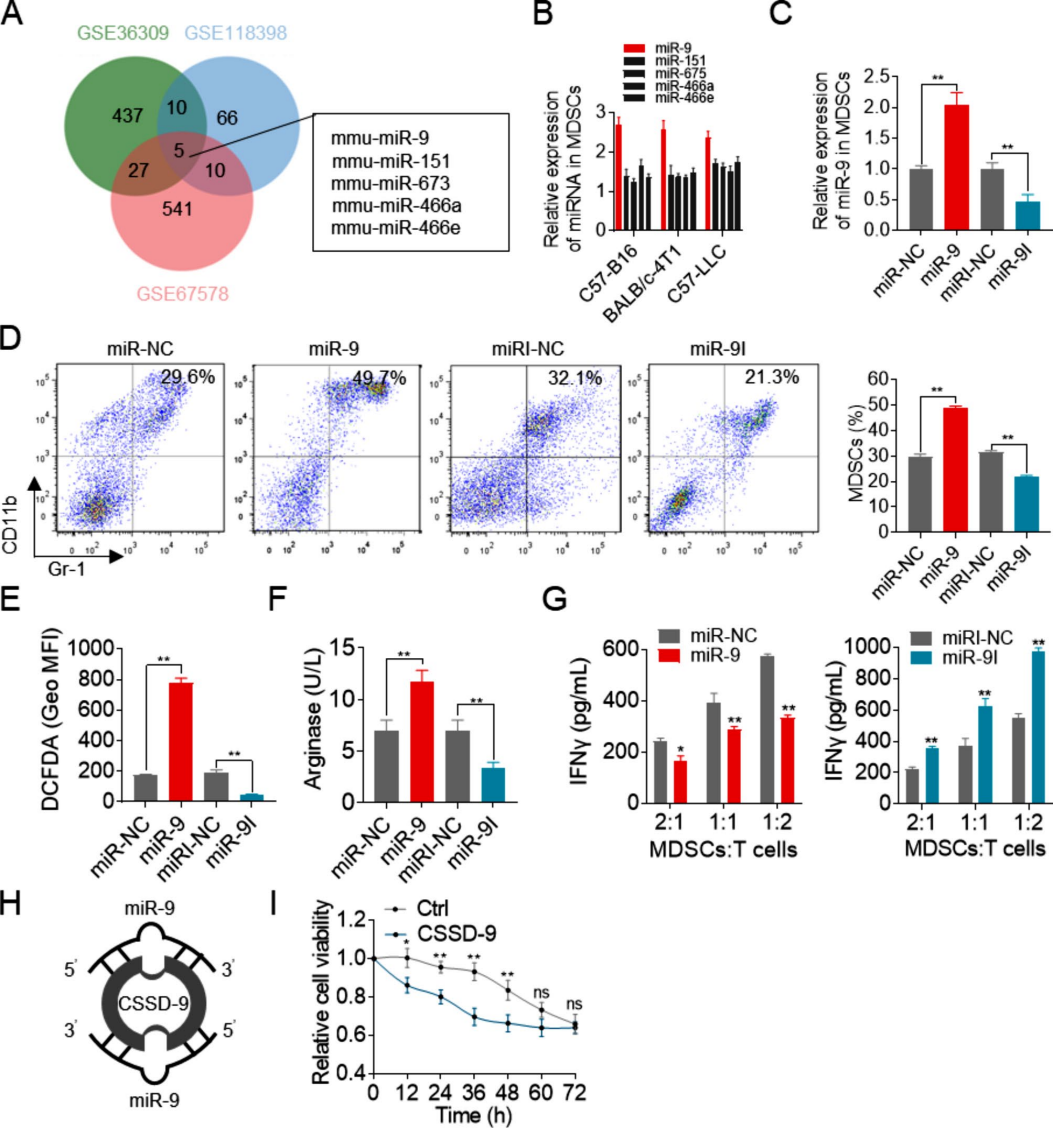
miR-9 in MDSCs improved its immunosuppressive function. (**A**) Venn diagram showing overlap between highly expressive microRNA in different models. (**B**) Relative expression of five microRNAs detected by qRT-PCR in different tumor-bearing models. U6 expression was used to endogenous control. (**C**) Relative expression of miR-9 in MDSCs measured by qRT-PCR. (**D**) MDSCs percentages detected in microRNA transfected BM cells by FACS. (**E**-**F**) ROS and Arginase activity levels measured with different treatments. MDSCs were transfected with miR-9 mimics or miR-9 inhibitors. After 24 h, cells were harvested and then cocultured with CD3^+ ^CD8^+^ T cells (MDSCs/T cells ratio of 2:1, 1:1, 1:2) for 72 h. (**G**) Immunosuppressive function of MDSCs measured by the level of IFNγ. (**H**) Structure of CSSD9. (**I**) Cell proliferation. *P < 0.05, **P < 0.01

To explore which miRNAs affect the induction and immunosuppressive functions of MDSCs, we retrieved miRNA expression datasets of MDSCs in three mouse models from the GEO database. It was noted that five miRNAs overlapped across three models (Fig. 1A). An analysis was carried out to determine the relative expression of the five common miRNAs. miR-9 was identified as the most significantly expressed miRNA (Fig. 1B). To investigate the effect of miR-9 on MDSCs induction, Bone Marrow cells (BM cells) were initially stimulated with GM-CSF/IL-6 for two days. Afterward, they were transfected with miR-9 mimics or inhibitors (Fig. 1C). Our results indicated that miR-9 overexpression increased the percentage of MDSCs from BM cells. Overexpression of miR-9 upregulated the percentage of MDSCs, and downregulation of miR-9 decreased the percentage of MDSCs (Fig. 1D). To assess the effect of miR-9 on the immunosuppressive function of MDSCs, arginase activity and reactive oxygen species (ROS) levels were examined. Our findings demonstrate that transfection of miR-9 mimics led to a significant increase in arginase activity and ROS levels. (Fig. 1E, F). To investigate whether MDSCs regulated by miR-9 inhibit T cell function, MDSCs were transfected with miR-9 mimics or inhibitors and were co-cultured with CD3 ^+^ CD8 ^+^ T cells for 48 h. The results showed that miR-9 observably increased the ability of MDSCs to suppress IFN-γ contents of T cell, whereas miR9-inhibitor alleviated MDSCs-mediated immunosuppressive function (Fig. 1G). Reducing miR-9 levels in MDSCs could improve the tumor microenvironment and decrease the immunosuppressive function of MDSCs in pathological conditions. CSSD9, a miR-9 inhibitor, was designed based on the sequence of mature miR-9. CSSD9 has two miR-9 binding sites and acts as miRNA sponges (Fig. 1H). To determine the effect of CSSD9 on the viability of MDSCs, we co-cultured MDSCs with nanoparticle-loaded CSSD-9. The MTT assay indicated that CSSD9 effectively inhibited the viability of MDSCs for 48 h (Fig. 1I). In order to prolong the activity and stability of CSSD9, we designed a new circular, single-stranded DNA with greater stability.

### G-quadruplex improves the stability of circular single-stranded DNA

**Fig. 2:**
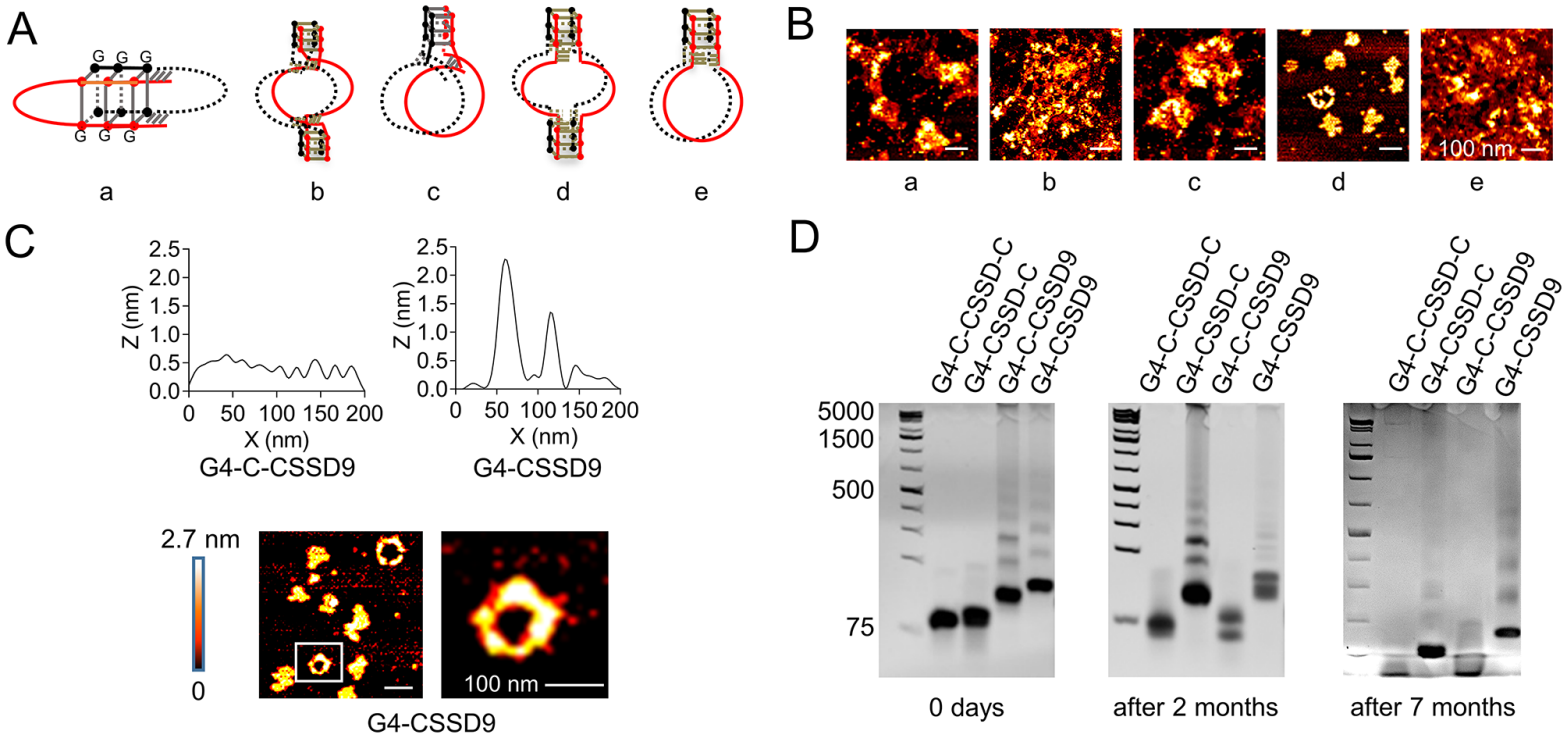
G-quadruplex improves the stability of CSSD9. (**A**) Designation of G4-CSSD9 connection way. (**B**) AFM images of different structural G4-CSSD9. (**C**) Self height difference between G4-C-CSSD9 and G4-CSSD9. (D) G4-CSSD9 structural stability detected by non-denaturing polyacrylamide gels

G-quadruplex (G4) is a highly stable four-stranded structure formed with guanine-rich (G-rich) nucleic acids. G4 is characterized by a stack of planar G-quartets, each of which contains four guanines connected by eight Hoogsteen hydrogen bonds. Monovalent cations can further stabilize G4 structure with a strength of in the order (K^+^> Na^+^> NH_4_^+^> Li^+^) [20]. Considering the high thermodynamic stability of G4 structures, a new stable circular single stranded DNA was designed (G4-CSSD9). In contrast to CSSD9, continuous guanine sequence (GGGGGGGGG) was added to 3’and 5’ ends. According to the connection of the base pair, we devised five different G4-CSSD9 (Fig. 2A, Table S1). The morphology and size were observed through atomic Force Microscope (AFM). The images indicated that the fourth connection G4-CSSD9 with ring shape and about 100 nm size was desired (Fig. 2B). The average height difference of the G4-CSSD9 was noticeably larger than that of the G4-C-CSSD9 (without continuous guanine sequence). As seen in the AFM image, we also found that G4-CSSD9 had a brighter area corresponding to the G-quadruplex (Fig. 2C). To investigate whether G-quadruplex could improve the stability of CSSD9, four different designed CSSD were performed in the non-denaturing polyacrylamide gels. We found that G4-C-CSSD-C and G4-C-CSSD9 could conform to different molecular weight bands at early stages. After two months, the bands were degraded. However, G4-CSSD9 obtained good stability that they were not degraded after seven months (Fig. 2D). To enhance the stability of G4-CSSD9 in vivo, we will use vesicles derived from MSCs for parcel delivery since MSCs are known for their low immunogenicity and can evade immune regulation.

### Preparation and characterization of MVs@G4-CSSD9

**Fig. 3:**
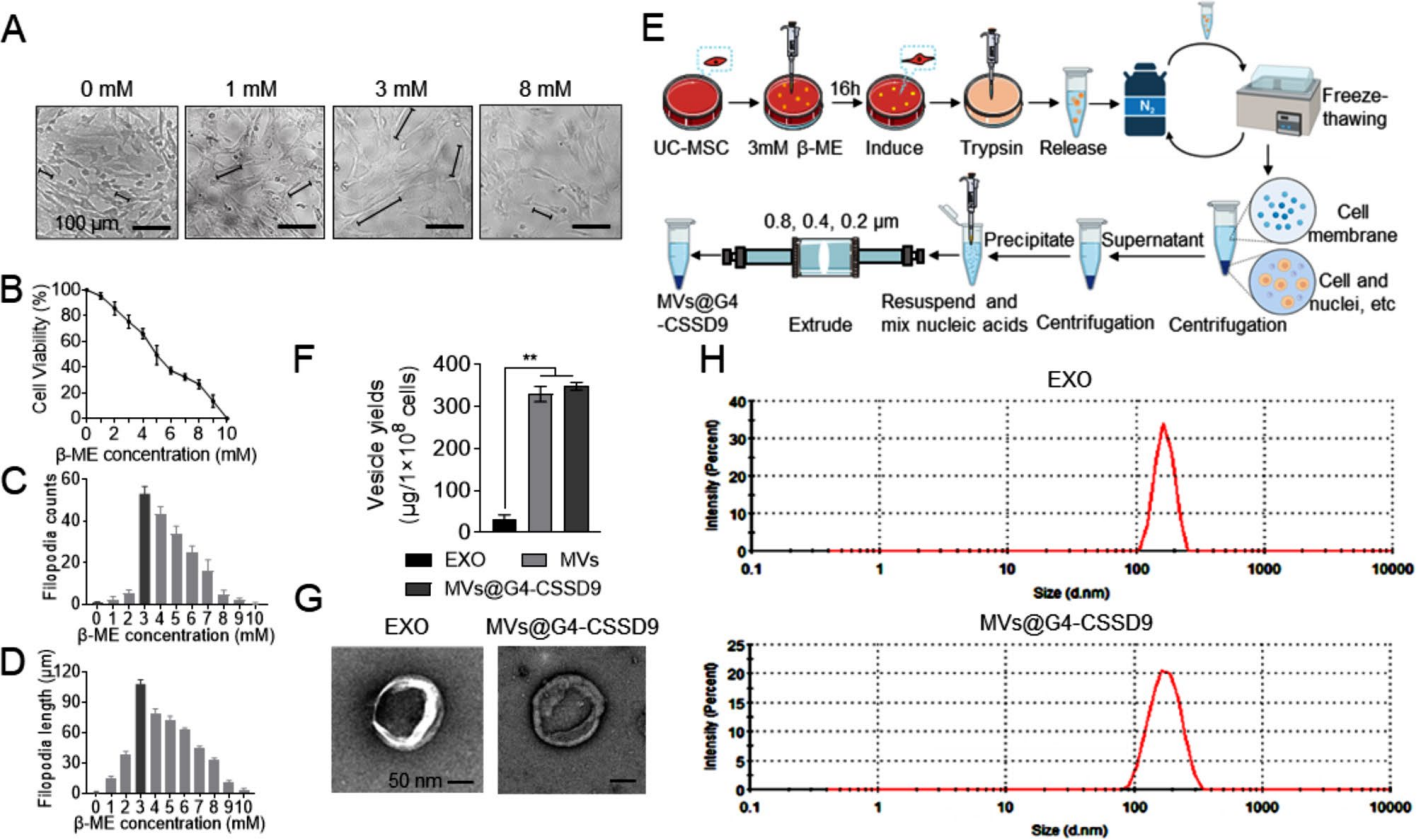
Preparation and characterization of MVs@G4-CSSD9. (**A**) Morphology, (**B**) cell viability, (**C**) filopodia’s numbers, and (**D**) length of UC-MSCs induced by different concentrations of β-ME. (**E**) Preparation process of MVs@G4-CSSD9. (**F**) Protein amount of EXO, MVs, and MVs@G4-CSSD9 from 1 × 10^8^ MSCs. (**G**) Transmission electron microscopy analysis of isolated EXO and MVs@G4-CSSD9 ultrastructural morphology. Scale bars: 100 nm. (**H**) Size distribution of EXO and MVs@G4-CSSD9 measured by Dynamic light scattering (DLS, Zetasizer Nano). *P < 0.05, **P < 0.01

To enhance the formation of filopodia, MSCs were induced using various concentrations of β-Mercaptoethanol (β-ME). An MTT assay was conducted 16 h after the treatment to assess the impact of β-ME on MSCs viability. The results demonstrated that 3 mM β-ME could enhance the count and length of filopodia, without impacting the cell viability. Nonetheless, high concentrations of β-ME caused significant cytotoxicity, leading to decreased cell viability (Fig. 3A-D). The preparation process of MVs@G4-CSSD9 is shown in Fig. 3E. We obtained 330 µg total protein of MVs with 1 × 10^8^ MSCs. We also obtained exosomes (EXO) from the culture medium using conventional ultracentrifugation. However, we found that the same number of cells produced only 30 µg of total protein of exosomes after culturing for 24 h (Fig. 3F). The Transmission Electron Microscope (TEM) images in Fig. 3G indicated no significant difference in morphology between MVs@G4-CSSD9 and EXO. Dynamic light scattering analysis of MVs@G4-CSSD9 showed a size distribution with a peak diameter of approximately 130–140 nm similar to the EXO (Fig. 3H).

### MVs@G4-CSSD9 could decrease the immunosuppressive function of MDSCs

**Fig. 4:**
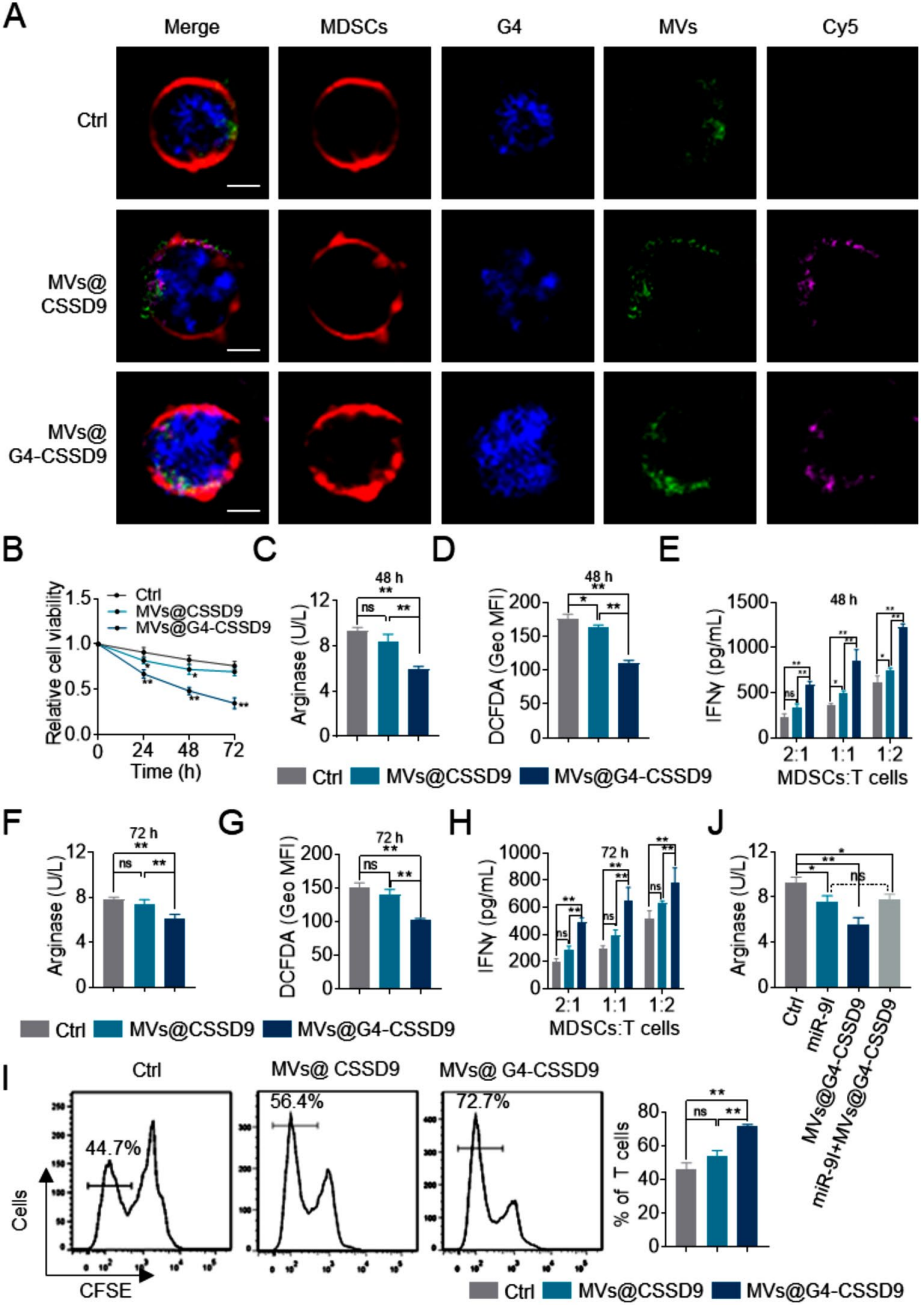
MVs@G4-CSSD9 could decrease the immunosuppressive function of MDSCs. (**A**) Confocal microscopy image of MDSCs with different treatment. Scale bar, 3 μm. (**B**) Proliferation detection of MDSCs with different treatment. (**C**-**E**) Arginase activity, ROS and IFNγ levels of MDSCs with different treatment for 48 h. (**F**-**H**) Arginase activity, ROS and IFNγ levels of MDSCs with different treatment for 72 h. (**I**) T cell proliferation measured by CFSE. (**J**) Arginase activity of MDSCs with different treatment. *P < 0.05, **P < 0.01

To detect whether the new drug carrier (MVs) could introduce CSSD9 into MDSCs, MVs@CSSD9 or MVs@G4-CSSD9 were co-cultured with MDSCs for 24 h. CSSD9 and G4-CSSD9 were marked with Cy5 (purple), the MVs were stained by PKH67 (green), and MDSCs were stained by PKH267 (red) in advance. As the images shown, confocal laser microscopy confirmed MVs co-localized with G4-CSSD9 in the membrane of MDSCs. The result suggested that MVs could introduce G4-CSSD9 into MDSCs and there was a natural targeting between MVs and MDSCs. MVs@CSSD9 or MVs@G4-CSSD9 were co-cultured with MDSCs, PKH26 labeled MDSCs (red) membrane and DAPI labeled nucleus. As the probe of G-quadruplex, IMT was used as a symbol that G4-CSSD9 was introduced into MDSCs. There was no difference between the group of MVs@CSSD9 and control. (Fig. 4A). To evaluate whether MVs@G4-CSSD9 could affect the cell viability and immunosuppressive function of MDSCs, series of experiments were examined. MTT assay showed that MVs@CSSD9 could impaired the viability of MDSCs for 48 h. Moreover, MVs@G4-CSSD9 could further prolong damage ability to MDSCs for 72 h (Fig. 4B). MVs@G4-CSSD9 further decreased arginase activity and ROS levels relative to MVs@CSSD9 (Fig. 4C-D and F-G). To determine the ability of MVs@G4-CSSD9 to suppress T cell activation and proliferation, T cells were co-cultured with MDSCs. The results indicated that MVs@G4-CSSD9 remarkably weakened the ability of MDSCs to suppress T cells and enhanced IFN-γ production relative to MVs@CSSD9 (Fig. 4E, H). In addition, G4-CSSD9 could further improve T cell proliferation by CFSE assay (Fig. 4I). Above indicated that MVs@G4-CSSD9 decreased the immunosuppressive capacity of MDSCs in vitro. To compare the immunosuppressive function MDSCs of miR-9I and MVs@G4-CSSD9, arginase level was examined. The results showed that miR-9I was worse than MVs@G4-CSSD9 for relieving immunosuppressive function of MDSCs. When both were introduced into the MDSCs, the suppression capacity remained unchanged (Fig. 4J). It suggested that MVs@G4-CSSD9 could not function when miR-9 inhibitor combined with miR-9 sequence.

### MVs@G4-CSSD9 inhibits tumor growth in vivo

**Fig. 5:**
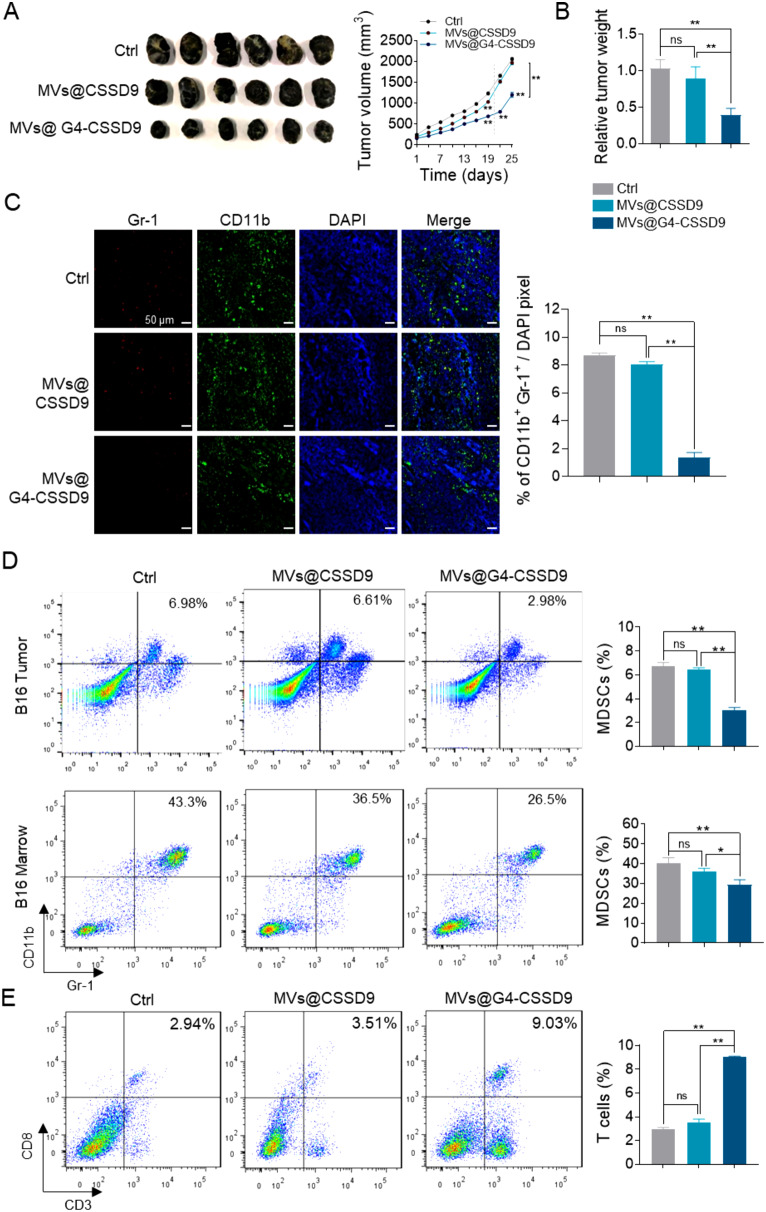
MVs@G4-CSSD9 inhibits tumor growth in vivo. (**A**) B16 tumor volumes and (**B**) relative tumor weight. (**C**) Representative pictures and quantitative data of tumor sections stained with Gr-1 (red), CD11b (green), and DAPI (blue). (**D**) MDSCs percentages detected in tumor and BM cells by FACS. (**E**) T cell percentages detected in spleen cells by FACS. *P < 0.05, **P < 0.01

Before efficacy testing in vivo, we preliminarily evaluated the safety of MVs@G4-CSSD9. The results indicated that the liver function and renal function were normal (Fig. S1). To investigate the inhibitory effects of G4-CSSD9 to tumor in vivo, about 1 × 10^6^ B16 cells were injected subcutaneously in the middorsal region of C57BL/6 mice. When the tumor volume reached 100 to 200 mm^3^, mice were treated with mixed MVs@G4-CSSD9 (20 µg) or MVs@CSSD9 (20 µg) and by intraperitoneal (I.P.) injection every 3 days to ascertain the dose dependency of the effect. Both MVs@G4-CSSD9 and MVs@CSSD9 exhibited antitumor effects during the administration period. However, MVs@G4-CSSD9 was more effective than MVs@CSSD9. Following discontinuation, MVs@G4-CSSD9 retained its antitumor effect, while MVs@CSSD9 lost its efficacy (Fig. 5A). In addition, we measured tumor weight. Compared with MVs@CSSD9 treatment, the administration of MVs@G4-CSSD9 showed more obvious antitumor effects (Fig. 5B). Immunofluorescence and FACS analyses revealed that MVs@G4-CSSD treatment significantly decreased the number of MDSCs (Fig. 5C and D). We isolated CD3^+^ CD8^+^ T cells from spleen. The percentage of T cells treated by MVs@G4-CSSD9 was significantly higher than control and MVs@CSSD9 group (Fig. 5E). The findings indicate that G4-CSSD9 potentially inhibits tumor growth in vivo by lowering the immunosuppressive activity of MDSCs upon MVs delivery.

## Discussion

In contrast to traditional drug carriers, such as liposomes, micelles, and polymeric nanoparticles, MVs as a natural drug carrier, can avoid phagocytosis and prolong the half-life of drugs in blood [21, 22]. It releases drugs into the cytoplasm through cell membrane fusion and has good biocompatibility [23]. However, the relative quantities of vesicles are released by cells. In this study, MSCs induced by 3 mM β-ME significantly increased the number and length of cellular filopodia. The treated MSCs were passed through extruders with different pore sizes to prepare MVs with similar shapes and sizes as exosomes and with significantly higher yields. MSCs have a wide range of sources and low immunogenicity [24, 25]. The vesicles prepared from it do not cause immune rejection into cells.

Different expressed microRNAs in MDSCs have been reported. Here, five miRNAs (mmu-miR-9, mmu-miR-151, mmu-miR-675, mmu-miR-466a, and mmu-miR-466e) were screened out in three different research models from GEO database. We tested the relative expressions of five microRNAs in MDSCs in vitro by qRT-PCR and found that miR-9 expression was the highest. Studies have reported that miR-9 could promote the immunosuppressive function [26]. Bone marrow cells were isolated from mice bone and transfected with miR9-mimics and miR9 inhibitors. miR-9 significantly increased the proportion of MDSCs in bone marrow cells, while miR-9 inhibitors decreased. Through testing some MDSCs functional indicators, it was found that miR-9 increased NO and arginase levels. After co-culturing MDSCs transfected with miR-9 mimics and T cells, IFNγ content was significantly reduced compared with the control. These results confirm the ability of miR9 to promote the immunosuppressive function of MDSCs. MicroRNA is a single-stranded non-coding RNA that is unstable and easily degraded. To improve its stability, we designed CSSD with reference to circRNA and constructed CSSD9 that specifically adsorbed the miR-9 sequence. We transfected CSSD9 into MDSCs and found that it can inhibit MDSCs activity for 48 h. There was no significant difference between the experimental group and the control after 48 h. We speculated that this may be related to the stability of CSSD9.

The G-quadruplex (G4) structure is an extremely stable quadruple-stranded DNA and RNA secondary structure held together by non-canonical guanine base pairs [27]. To further improve the stability of CSSD9, we added G4 to both ends of the sequence when designing the cyclic single-stranded DNA. In nondenaturing polyacrylamide gels, G4-CSSD9 could form cyclic structures with different molecular weights and could be maintained for a long time. In vitro experiments showed that the antitumor effect of MVs@G4-CSSD9 lasted longer compared with MVs@CSSD9. Further in vivo experiments showed that after the end of administration, MVs@CSSD9 was unable to exert anti-tumor effects by deregulating the immunosuppressive activity of MDSCs leading to further rapid tumor growth, whereas MVs@G4-CSSD9 was still effective in deregulating immunosuppression and anti-tumor. This may be attributed to the G4 structure conferring good stability to the nucleic acid drug, which is not easily decomposed in vivo. These results suggest that G4 can actually enhance the stability of CSSD9 and exert excellent anti-tumor effects.

## Conclusions

In conclusion, we designed an artificial DNA called G4-CSSD9 that specifically adsorbs miR-9 and has good stability. Meanwhile, we induced MSCs to produce a large number of filamentous pseudopods with β-ME, which in turn led to the use of extrusion to obtain MVs with similar properties to conventional exosomes and greatly improved the yield. The G4-CSSD9 encapsulated by MVs can effectively target miR-9 and relieve the immunosuppressive function of MDSCs for the treatment of melanoma.

### Cell culture and transfection

B16 cells were cultured in DMEM medium (HyClone, USA), and UC-MSCs were cultured in DMEM/F12 medium (HyClone, USA) supplemented with 10% (v/v) fetal bovine serum (Thermo Fisher Scientific, USA) at 37 °C in a humidified atmosphere containing 95% air and 5% CO_2_. All cells were purchased from KeyGen Biotech (Nanjing, China) and the company provides complete cell identification. MDSCs were cultured in RPMI-1640 medium with GM-CSF and IL-6. miR-9 mimics, miR-9 inhibitors, miR-NC and inhibitor control were synthesized by RiboBio (Guangzhou, China). The control used for the miR-9 mimics was miR-NC. The control used for miR-9 inhibitor was inhibitor-control. Oligonucleotide transfection was performed using a Micropoly-transfecter™ Cell Reagent (Biosky, China) according to the manufacturer’s instructions. Briefly, MDSCs were seeded in a 24-well plate with complete growth medium. The Micropoly core transfection reagent and transfection booster were added and mixed thoroughly. DNA were added to the Micropoly reagents and mixed by gentle pipetting. The transfection mixture was incubated at room temperature for 15 min before it was added to the cells in a drop-wise manner. Cells were incubated for 72 h at 37 °C.

### Cell proliferation assay

Cells were seeded in a 96-well plate (5000 cells per well). At 12, 24, 36, 48, 60, and 72 h after CSSD9 (0.5 µg) treatment, 20 µL of MTT (5 mg/mL) was added. Then, the MDSCs were incubated for 4–6 h at 37 °C in 5% CO_2_. The supernatant solution was discarded, adding 100 µL dimethyl sulfoxide to each well. Absorbance was detected at a wavelength of 490 nm using the Multiskan™ FC Microplate Photometer (Thermo Scientific, USA). The measurement was repeated at least three times.

### G4-CSSD9 preparation

Annealing buffer: 5×K^+^ annealing buffer (100 mM Tris-HCl, 750 mM KCl, 15 mM MgCl_2_) mix with 5×Annealing buffer for DNA oligos (Beyotime, China) to 1:1, then diluted to 1×with G4 buffer (10 mM Tris-HCl PH = 7.5, 1 mM MgCl_2_). Annealing procedure: 95 °C, 5 min; 25 °C, 90 min. The primers were shown in Table S2.

### Cell nanovesicles preparation

EXO preparation: Exosomes were obtained using gradient centrifugation. Briefly, the culture supernatant of MSCs was collected and aspirated into a 50 mL centrifuge tube and centrifuged at 300×g for 10 min at 4 °C to remove the cellular precipitate. The supernatant was collected and centrifuged at 2000×g for 10 min at 4 °C to remove the dead cell precipitate. The supernatant was collected and centrifuged at 10000×g for 30 min at 4 °C to remove cell debris. Next, the supernatant was filtered through a 0.22 μm membrane into another ultra-high speed centrifuge tube. The resulting supernatant was further centrifuged at 100000×g for 70 min at 4 °C to collect the exosome precipitate. Wash the exosome precipitate with an equal amount of PBS and repeat the previous step; discard the supernatant, collect the exosome precipitate and store at -80 °C.

MVs@G4-CSSD9 preparation: The liposome extruder and polycarbonate filter membrane were immersed in 75% ethanol for 30 min, followed by exposure to ultraviolet light for 30 min on a clean bench. Cell membranes were extracted from β-ME induced cells for 16 h, centrifuged, and resuspended in membrane protein extraction reagent A (Beyotime, China). The suspension was placed in an ice bath for 15 min, subjected to repeated freeze-thaw cycles between liquid nitrogen and a 37 °C water bath, repeated three times. The suspension was centrifuged at 4 °C, 700×g for 10 min, and the supernatant was collected. The precipitate inside the tube was discarded, and the remaining pellet consisted of cell nuclei and undamaged cells. The supernatant was centrifuged at 4 °C, 14000×g for 30 min, and the resulting precipitate was the tumor cell membrane. The cell membrane was resuspended in PBS and combined with G4-CSSD9. The suspension was transferred to the liposome extruder, and extrusion was performed through polycarbonate filter membranes of different pore sizes (0.8, 0.4, and 0.2 μm) in ascending order, with each pore size subjected to three extrusions. MVs@G4-CSSD9 were collected and stored at -80 °C.

### Nanovesicles characterization

Electron microscopy was taken with a transmission electron microscopy to obtain morphology information. Particle size was detected using a nanoparticle size analyzer (nano ZS, UK).

### Comparison of EXO and MVs production

The BCA protein quantification method was used to compare vesicle yields obtained by conventional gradient centrifugation and extrusion methods by the liposome extruder for the same cell number (1 × 10^8^).

### Animals and establishment of tumor-bearing mouse models

Female C57BL/6 mice, 4–6 weeks, were purchased from the department of SiPeiFu (Beijing, China) and were housed under specific pathogen-free (SPF) conditions at least 1week before use. Subcutaneous tumor models were established by injecting 1 × 10^5^ B16 cells subcutaneously into the flank. The weight and tumor size of mice was measured to monitor tumor growth. Drugs will be administered every three days, discontinued on day 21 after initiation of administration, and observation will continue for four days after discontinuation, after which the mice will be put to death for further analysis. All animal experiments were performed in accordance with animal use and handling protocols.

### CD11b^+^ Gr-1^+^ MDSCs isolation

Mice bone marrow singlecell suspensions were prepared were dissociated mechanically and individual marrow cells were obtained through a 75-µm cellular sieve, and then centrifuged at 2000 rpm for 10 min at 4˚C. Red blood cells were removed using ACK Lysis Buffer (Leagene Biotech, China) in accordance with the manufacturer’s instructions. CD11b Cells were washed with PBS and counted. Single-cell suspensions (1 × 10^6^- 1 × 10^8^) prepared from the marrow were stained with the following specific fluorophore-conjugated anti-mouse antibodies CD11b and Gr-1 in PBS for 40 min in the dark at 4 °C. The stained cells were washed with PBS. The CD11b^+^ Gr-1^+^ MDSCs were purified using flow cytometer (BD Biosciences, USA).

### Isolation of T cells

After euthanasia, mice were immersed in 75% ethanol for 3 min, and then the spleens were isolated using sterilised surgical equipment. Splenic tissue was minced into small pieces with sterile scissors and gently pressed through a 100-µm mesh cell strainer with a syringe piston. Add 1 mL of erythrocyte lysate to the EP tube containing spleen cells, lysed for 10 min at room temperature, centrifuged to collect the cell precipitate, washed twice with PBS, resuspended in PBS solution, and counted by a cell counter. The CD3 and CD8 antibody (Sungene Biotech, China) labeling working solution was prepared by diluting the antibodies in PBS to a concentration of 2.5 µg/mL. 1 × 10^7^cells were then stained with the prepared working solution, washed with PBS, and resuspended. Subsequently, the stained cells were placed in a flow tube and analyzed and sorted using a flow cytometer.

### Quantitative real-time PCR (qRT-PCR)

Total RNA was extracted from MDSCs cells using TRIzol reagent (Invitrogen). RNA concentration and purity were determined through measurement of A260/280 ratios with Nanodrop. cDNAs were synthesized from total RNA using the PrimeScript RT Reagent Kit (Tiangen, China). U6 was used as an internal control. A SYBR RT-PCR kit (Tiangen, China) was used for transcript quantification with specific primers and the relative expression levels were calculated using the 2^−ΔΔCt^ method. Each sample was assessed in triplicate.

### AFM imaging

The annealed samples were diluted with G4 Buffer to 5 μm and placed on the mica surfaces. It was dried at room temperature and washed three times with Milli-Q water. Imaging was performed with atomic force microscopy. The images were analyzed and visualized using NanoScope Analysis software.

### DNA stability assay

In order to verify that G-quadruplex can enhance the stability of cyclic single-stranded DNA, non-denaturing gel was used to detect the band size distribution of four different cyclic single-stranded DNA after electrophoresis at different times. Briefly, DNA was extracted with phenol-chloroform, and the extracted DNA samples were added into the wells of the non-denaturing gel. The voltage was set to 90 V and electrophoresis was carried out on ice for 70 min. The gel was stained in TBE electrophoresis buffer containing nucleic acid dye for 30 min, washed three times and imaged. The gel was then reexamined at 2 months and 7 months, respectively.

### ROS measurements

MDSCs were seeded into a 6-well plate (1 × 10^6^ cells per well) and cultured for 12 h. Subsequently, they were treated with MVs@CSSD9 (CSSD9, 8 µg) and MVs@G4-CSSD9 (G4-CSSD9, 8 µg) for 48 h. Cells were then harvested by centrifugation and incubated with 1.0 µM DCFDA (Invitrogen, USA) at 37 °C for 20 min. After washing twice with PBS, cells were resuspended and analyzed for ROS levels through flow cytometry.

### Arginase and IFNγ measurements

MDSCs were seeded in a 96-well plate (5000 cells per well) and cultured for 12 h. Subsequently, they were treated with MVs@CSSD9 (CSSD9, 0.5 µg) and MVs@G4-CSSD9 (G4-CSSD9, 0.5 µg) for 48 h.

Arginase activity was measured using the Arginase Activity Assay Kit (Abcam, UK). A 100 µL aliquot of the lysis buffer was used to lyse cells in a 96-well plate for the control group, MVs@CSSD9 group, and MVs@G4-CSSD9 group. Target samples were incubated for 20 min at 37 °C with H_2_O_2_ substrate solution, while background wells were incubated with additional buffer. Standards were prepared following the kit instructions, and the enzymatic reaction mixture was added to all wells. Raw absorbance values were immediately obtained over a 30 min period at OD = 570 nm using a Multiskan™ FC Microplate Photometer (Thermo Scientific, USA). Arginase Activity Units were then calculated from raw absorbance values. ΔOD (ΔOD= (OD_2_-OD_BG2_)-(OD_1_-OD_BG1_)) was used to obtain the nmol of H_2_O_2_ generated by arginase, collected from a standard curve of known H_2_O_2_ concentrations. Arginase activity is calculated as (B/ΔT*V) *D in units/mL, where B is amount of H_2_O_2_ from standard curve (nmol), V is the sample volume added into reaction well (µL), D is sample dilution factor and ΔT is the reaction time in min. One unit of Arginase activity refers to the amount of arginase that will generate 1.0 nmol of H_2_O_2_ per min at pH 8 at 37 °C.

IFNγ levels were detected using the IFNγ ELISA kit (keyGEN, China). Culture supernatants from each group were collected and added to a microplate coated with anti-human IFNγ antibody. Human IFNγ standard and the test samples were added, followed by the addition of another biotinylated anti-human IFNγ antibody (HRP-conjugated antibody). After incubation and thorough washing at 37 °C, the color substrate was added. The color change, indicating the presence of IFNγ, was measured at 450 nm using a Multiskan™ FC Microplate Photometer (Thermo Scientific, USA). The OD values were correlated with the concentration of IFNγ in the samples, calculated by fitting a standard curve.

### T cell proliferation detection

The T cell proliferation was assessed using the CFSE Cell Proliferation Assay Kit (Bestbio, China). Following the manufacturer’s instructions, a staining working solution was prepared by diluting the CFSE stock solution 500 times with cell labeling solution, and then further diluting the resulting solution 2 times with PBS solution. This prepared staining working solution was used to resuspend T cells in a total volume of 1 milliliter, and the suspension was incubated at 37 °C for 30 min. Afterward, the cells were centrifuged at 1200 rpm for 10 min, the supernatant was discarded, and the cells were washed with PBS before being resuspended. MVs@CSSD9 and MVs@G4-CSSD9 were co-cultured with MDSCs for 72 h, followed by the separation of MDSCs. Subsequently, MDSCs were co-cultured with T cells at ratios of 2:1, 1:1, and 1:2 for 24 h. Fluorescence intensity was then assessed using flow cytometry.

### Immunofluorescence

Cy5 labeling was employed for the synthesis of CSSD9/G4-CSSD9. MVs were stained with PKH67, and MDSCs cell membranes were stained with PKH26. MDSCs cell membranes were pre-stained with PKH26. The PKH26 probe was diluted 500 times in the dilution solution provided in the reagent kit to prepare the staining working solution. MDSCs cells (1 × 10^7^ cells) were centrifuged to collect the cell pellet, washed with PBS, and then centrifuged again. The cell pellet was resuspended in the PKH26 staining working solution, incubated at 25 °C for 5 min, with gentle inversion of the centrifuge tube to ensure thorough mixing. An equal volume of serum was added to stop the staining reaction, incubated for 1 min, and the cells were centrifuged at 400×g for 10 min. The supernatant was carefully aspirated and discard. The cells were resuspended in 10 mL complete culture medium, transferred to another sterile centrifuge tube, and centrifuged at 400×g for 5 min at 25 °C. The cells were washed twice with 10 mL complete culture medium to remove unbound dye. Finally, the cells were resuspended in 10 mL complete culture medium and seeded in a 24-well cell culture plate with climbing slides. The MVs preparation was stained with PKH67 according to the manufacturer’s instructions. PKH67 probe was diluted 250 times in serum-free culture medium to prepare the staining working solution, ensuring sufficient coverage of the sample range. Incubation was carried out at 37 °C for 5 min, followed by a 15 min incubation at 4 °C. The precipitate was then collected by centrifugation, washed twice with PBS, and finally resuspended in PBS. Subsequently, MVs@CSSD9 or MVs@G4-CSSD9 were co-incubated with MDSCs for 48 h. After co-culturing, the samples were labeled with the G4 probe. After washing with PBS, they were transferred to a confocal chamber for imaging using laser confocal microscopy.

### Statistical analysis

Statistical analysis was performed using GraphPad Prism 8. Statistically significant differences were calculated using Student’s *t*-test, one-way ANOVA, and two-way ANOVA. *P* < 0.05 were considered to indicate a statistically significant difference.

## Electronic supplementary material

Below is the link to the electronic supplementary material.

Supplementary Material 1

## Data Availability

The datasets used and/or analyzed during the current study are available from the corresponding author on reasonable request.
