# Correction: MSC microvesicles loaded G-quadruplex-enhanced circular single-stranded DNA-9 inhibits tumor growth by targeting MDSCs

**DOI:** 10.1186/s12951-025-03313-1

**Published:** 2025-03-26

**Authors:** Jingxia Han, Rong Qin, Shaoting Zheng, Xiaohui Hou, Xiaorui Wang, Huihui An, Zhongwei Li, Yinan Li, Heng Zhang, Denghui Zhai, Huijuan Liu, Jing Meng, Tao Sun

**Affiliations:** 1 State Key Laboratory of Medicinal Chemical Biology and College of Pharmacy, Nankai University, Tianjin, China; 2 State Key Laboratory of Food Nutrition and Safety, Tianjin University of Science and Technology, Tianjin, China; 3 Precision Medicine Center, The First Affiliated Hospital of Xi’an Jiaotong University, Xi’an, China; 4 College of Life Sciences, Nankai University, Tianjin, China

**Correction: Journal of Nanobiotechnology (2024) 22:237**

10.1186/s12951-024-02504-6.

In this article Fig. 2 appeared incorrectly and have now been corrected in the original publication. For completeness and transparency, the incorrect and correct versions of Fig. 2 are displayed below.

Incorrect Fig. 2.

**Fig. 2:**
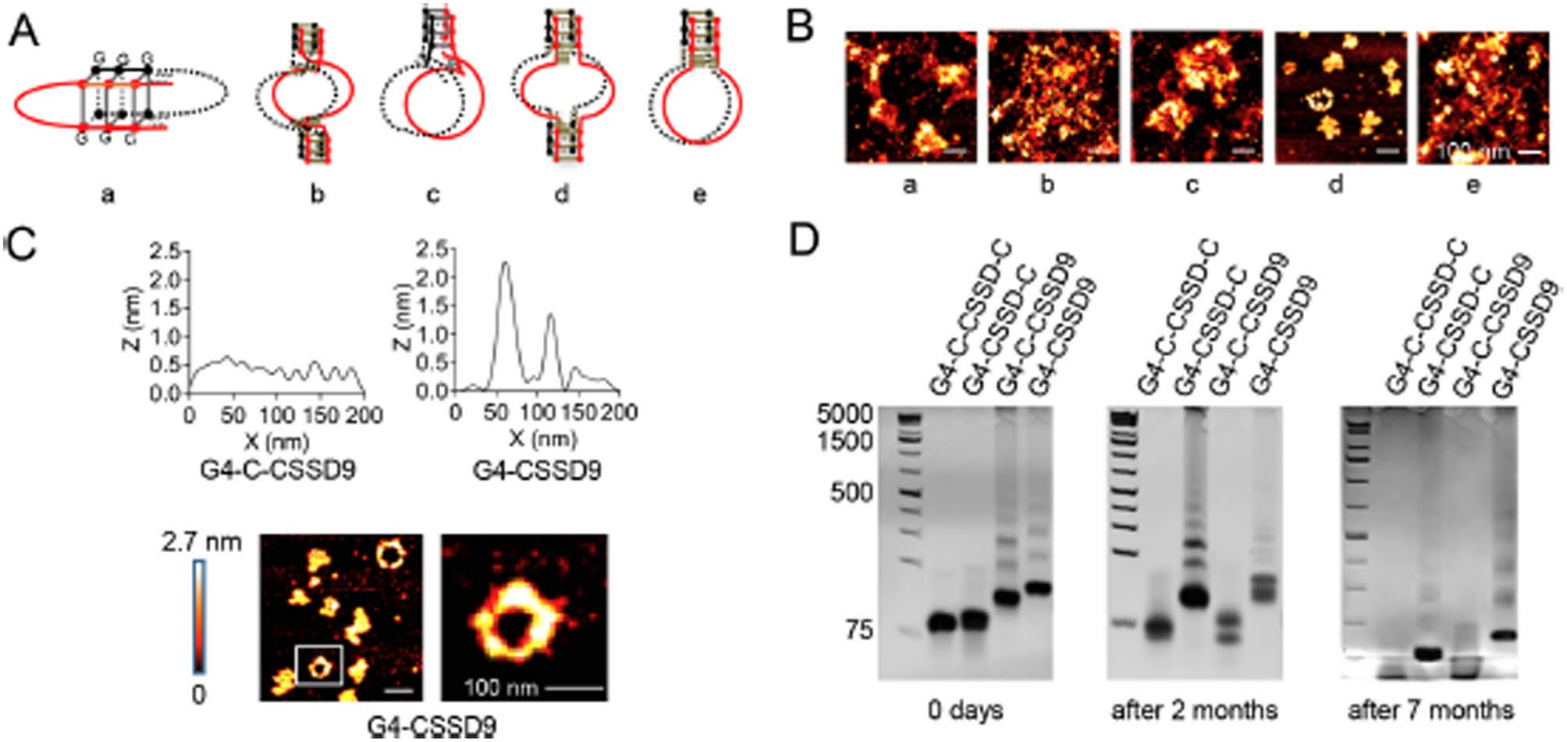
G-quadruplex improves the stability of CSSD9. (**A**) Designation of G4-CSSD9 connection way. (**B**) AFM images of different structural G4-CSSD9. (**C**) Self height difference between G4-C-CSSD9 and G4-CSSD9. (**D**) G4-CSSD9 structural stability detected by non-denaturing polyacrylamide gels

Correct Fig. 2.

**Fig. 2:**
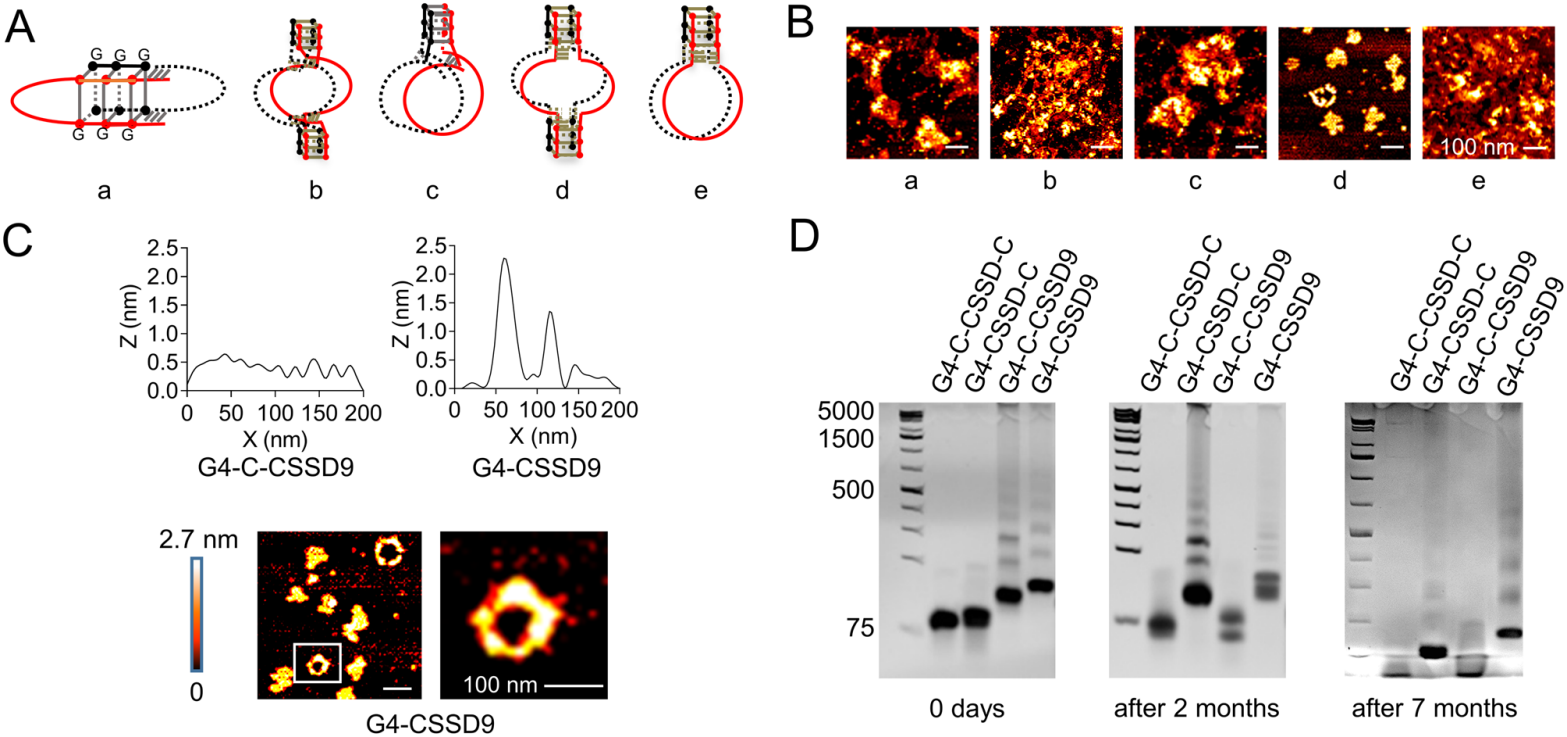
G-quadruplex improves the stability of CSSD9. (**A**) Designation of G4-CSSD9 connection way. (**B**) AFM images of different structural G4-CSSD9. (**C**) Self height difference between G4-C-CSSD9 and G4-CSSD9. (**D**) G4-CSSD9 structural stability detected by non-denaturing polyacrylamide gels

The original article has been corrected.

