# Supplementary material for: MSC microvesicles loaded G-quadruplex-enhanced circular single-stranded DNA-9 inhibits tumor growth by targeting MDSCs
Source: J Nanobiotechnology. 2024 May 12;22:237. doi: 10.1186/s12951-024-02504-6 (PMC11089713; doi:10.1186/s12951-024-02504-6)
Supplement: Supplementary file 1 — Supplementary Material 1 [file 12951_2024_2504_MOESM1_ESM.pdf]

Table S1. Five different designment of G4-CSSD9.

Gene	Sequence（5'→3'）
a	GGGGGGGGGGTTCAAACCGCTCATACAGCTAGATAACCAAAGACCATCTTCAAC<br>AATGGGGGGGGG<br>GCGGTTTGAACGGGGGGGGGTCATACAGCTAGATAACCAAAGAGGGGGGGGGA<br>TTGTTGAAGATGG
b	GGGGGGGGGAGTTGTTCAAACCGCTCATACAGCTAGATAACCAAAGACCATCTT<br>CAACAATTGATGGGGGGGGG<br>GGGGGGGGGAGTTGCGGTTTGAACTCATACAGCTAGATAACCAAAGAATTGTTG<br>AAGATGGTGATGGGGGGGGG
c	GGGGGGGGGAGTTATTGTTGAAGATGGTCATACAGCTAGATAACCAAAGATAACA<br>ACTTCTACCTGATGGGGGGGGG
d	GGGGGGGGGAGTTGTTCAAACCGCTCATACAGCTAGATAACCAAAGACCATCTT<br>CAACAATTGATGGGGGGGGG<br>GGGGGGGGGAGTTCAAGTTTGGCGTCATACAGCTAGATAACCAAAGAGGTAGAA<br>GTTGTTATGATGGGGGGGGG
e	GGGGGGGGGAGTTATTGTTGAAGATGGTCATACAGCTAGATAACCAAAGACCAT<br>CTTCAACAATTGATGGGGGGGGG

Table S2. The primers of G4-CSSD.

Gene	Primer（5'→3'）
G4-C-CSSD-C-T	AGTTAGTTAAGTTGTTCAAACCGCACGTGACACGTTCGGAGAATTCCATCTTCAAC<br>AATTGATTGCAGCGAT
G4-C-CSSD-C-B	AGTTAGTTAAGTTCAAGTTTGGCGACGTGACACGTTCGGAGAATTGGTAGAAGTT<br>GTTATGATTGCAGCGAT
G4-CSSD-C-T	GGGGGGGGGAGTTGTTCAAACCGCACGTGACACGTTCGGAGAATTCCATCTTCA<br>ACAATTGATGGGGGGGGG
G4-CSSD-C-B	GGGGGGGGGAGTTCAAGTTTGGCGACGTGACACGTTCGGAGAATTGGTAGAAGT<br>TGTTATGATGGGGGGGGG
G4-C-CSSD9-T	AGTTAGTTAAGTTGTTCAAACCGTCATACAGCTAGTAACCAAAGACCATCTTCAACA<br>ATTGATTGCAGCGAT
G4-C-CSSD9-B	AGTTAGTTAAGTTCAAGTTTGGCTCATACAGCTAGTAACCAAAGAGGTAGAAGTTG<br>TTATGATTGCAGCGAT
G4-CSSD9-T	GGGGGGGGGAGTTGTTCAAACCGTCATACAGCTAGTAACCAAAGACCATCTTCAA<br>CAATTGATGGGGGGGGG
G4-CSSD9-B	GGGGGGGGGAGTTCAAGTTTGGCTCATACAGCTAGTAACCAAAGAGGTAGAAGTT<br>GTTATGATGGGGGGGGG

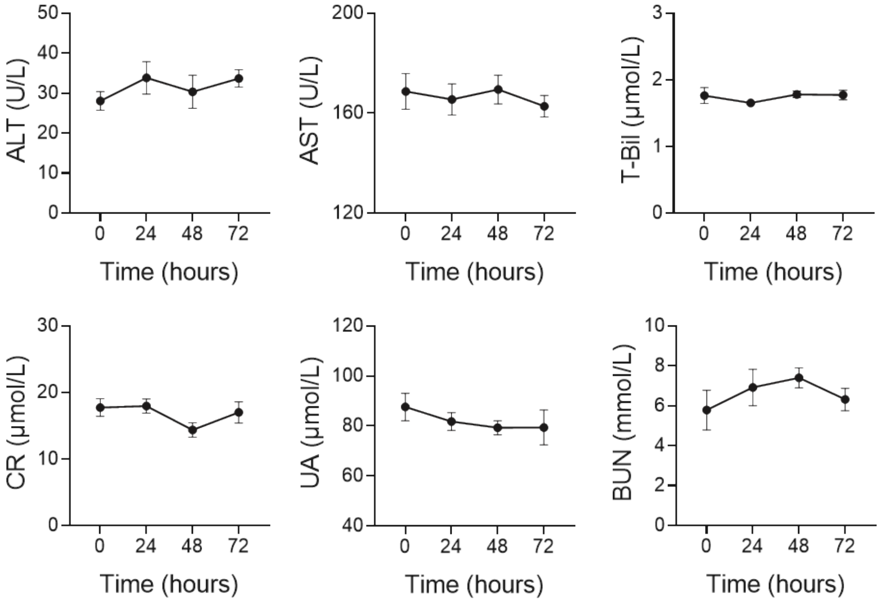

Fig. S1. Biochemical analysis of liver function (ALT, AST, and T-Bil) and kidney function (CR, UA, and BUN) in mice.
